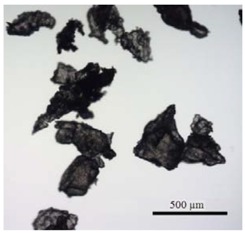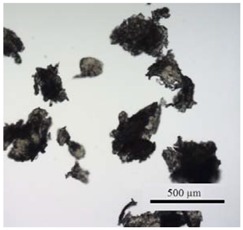# Biodegradable and Petroleum-Based Microplastics Do Not Differ in Their Ingestion and Excretion but in Their Biological Effects in a Freshwater Invertebrate *Gammarus fossarum*

**DOI:** 10.3390/ijerph14070774

**Published:** 2017-07-13

**Authors:** Sandrine Straub, Philipp E. Hirsch, Patricia Burkhardt-Holm

**Affiliations:** Man-Society-Environment (Program MGU), Department of Environmental Sciences, University of Basel, Vesalgasse 1, 4051 Basel, Switzerland; sandrinestraub@hotmail.com (S.S.); philipp.hirsch@unibas.ch (P.E.H.)

**Keywords:** microplastic pollution, environmental effects, bioplastic, amphipoda

## Abstract

Research on the uptake and effects of bioplastics by aquatic organisms is still in its infancy. Here, we aim to advance the field by comparing uptake and effects of microplastic particles (MPP) of a biodegradable bioMPP (polyhydroxybutyrate (PHB)) and petroleum-based MPP (polymethylmethacrylate (PMMA)) in the freshwater amphipod *Gammarus fossarum*. Ingestion of both MPP in different particle sizes (32–250 µm) occurred after 24 h, with highest ingestion of particles in the range 32–63 µm and almost complete egestion after 64 h. A four-week effect-experiment showed a significant decrease of the assimilation efficiency in amphipods exposed to the petroleum-based MPP from week two onwards. The petroleum-based PMMA affected assimilation efficiency significantly in contrast to the biodegradable PHB, but overall differences in direct comparison of MPP types were small. Both MPP types led to a significantly lower wet weight gain relative to the control treatments. After four weeks, differences between both MPP types and silica, used as a natural particle control, were detected. In summary, these results suggest that both MPP types provoke digestive constraints on the amphipods, which go beyond those of natural non-palatable particles. This highlights the need for more detailed research comparing environmental effects of biodegradable and petroleum-based MPP and testing those against naturally occurring particle loads.

## 1. Introduction

### 1.1. Microplastic Particles as an Environmental Issue

The promise of biodegradable bioplastics is that they put an end to the accumulation of plastic in the environment. Lately, however, the issue of plastic in the environment is not only that of accumulation but also that of direct environmental threats originating from microplastics (MP < 5 mm) [1]. Microplastic particles (MPP) can be found in nearly every aquatic ecosystem and even in remote areas such as deep-sea sediments [2] and the Arctic sea-ice [3]. Microplastic particles concentrations, as well as the prevalent MPP types depend on the sampling site (e.g., surface water, water column or sediments) and on the method of sampling (reviewed in [4]). Recent studies found MPP to be prevalent in freshwater ecosystems in high abundances. For example, a recent study in the River Rhine found an average of 892,777 particles km^−2^ which highlights the potential environmental relevance of MPP pollution in freshwater systems [5].

### 1.2. Effects of Petroleum-Based and Biodegradable Microplastic Particles

Upon entrance into the environment, MPP can become bioavailable and can thus cause environmental effects. Effects of conventional MPP are mainly studied along two lines: investigations on the uptake by biota and investigations on the pertinent effects following the uptake. The uptake of these substances depends on several factors including the size of the particles in relation to the size of the regarded species and the density of the MPP (reviewed in [6,7]). The biological effects are mostly described in the form of direct interference with food uptake and physical effects on gastrointestinal cavities. In general, laboratory experiments showed reduced food consumption [8,9], reduced energetic reserves for growth [10], a reduced survival potential [11], and reduced fitness [12]. Compared to petroleum-based MPP, studies on bio-based and biodegradable MPPs have been rather scarce. The few works published up to the present day have been conducted in marine environments and demonstrated that both biodegradable and petroleum-based MPP can affect the function of marine bottom-living organisms: The sandworm *Arenicola marina* [13] and the European flat oyster *Ostrea edulis* [14] both changed their feeding and filtering behavior in mesocosm experiments. No significant differences between MPP types were detected. However, these studies were conducted under uncontrolled outdoor conditions, which did not allow for a detailed investigation for MPP-type-dependent ingestion and excretion. To our knowledge there is still no study comparing the ingestion and excretion and subsequent biological effects of biodegradable and petroleum-based MPP under controlled laboratory conditions.

### 1.3. Research Gap

If bioplastics are to be developed as an alternative to petroleum-based plastics, then we need more comparable data on their direct effects on organisms. This research need is especially imminent because aquatic ecosystems and their biota provide key ecosystem services to humans [15]. The existing research on biodegradable and petroleum-based MPP effects on marine organisms needs to be complemented by a more detailed understanding of the effects of both MPP types on key freshwater organisms.

### 1.4. Study Aim, Study System and Study Organism

Our study aimed to test whether biodegradable and petroleum-based MPP differ in their effects on an organism representing an ecologically relevant freshwater feeding guild. To address this question we compared the ingestion, egestion, and the biological effects of a bio-based and biodegradable bioMPP polyhydroxybutyrate (PHB) and a petroleum-based conventional MPP polymethylmethacrylate (PMMA) in the freshwater amphipod *Gammarus fossarum*. The MPP types were selected based on similarities in physical properties such as size, sphericity, specific density, and in their pathways into the environment. Polyhydroxybutyrate is the most common member of the biopolymer group polyhydroxyalkanoates (PHA) and is biosynthesized by microbes, primarily as a form of energy storage molecule [16]. At least 75 different genera of microorganisms are able to produce PHB, and more than 100 different monomer units have been identified so far [17]. Polymethylmethacrylate is the polymer of methyl methacrylate (MMA); it is widely used and also known as acrylic glass [18]. Recent reviews have highlighted the contribution of personal care products to microplastic pollution in the environment [19]. Both MPP are used in the personal care industry and therefore constitute realistic sources of pollution entering the natural aquatic environment as primary MPP.

*Gammarus fossarum* was used as a study organism because it is a major component of temperate freshwater food webs. As a member of the feeding guild of shredders it chews and grinds its food and thus plays an important role in the decomposition of coarse particulate organic matter, e.g., plant litter in freshwater ecosystems [20]. It is a bottom-living, i.e., benthic, organism at the base of many temperate freshwater food webs [20]. Its functions for nutrient and organic matter cycling are well-studied and it is frequently used as a model organism for ecotoxicological studies [21]. We therefore assumed that *Gammarus fossarum* would be a relevant organism, whose feeding ecology and habitat would expose it to MPP entering the freshwater environment. 

### 1.5. Experimental Approach and Hypotheses

Our experimental approach aimed to compare the uptake and the effects of both MPP within the study organism under controlled laboratory conditions. In a first experiment, we therefore explored the ingestion and excretion of both MPP in standardized particle sizes and concentrations based on previous research [22]. In a second long-term (28 days) experiment, we tested the biological effects of the particles on three biomarkers: feeding rate, assimilation efficiency, and weight change. Feeding rate (FR) in amphipods is a biomarker for how the uptake of food changes in response to toxic substances associated with the food source [23]. In our case, we do not expect any of the particles to be toxic and therefore hypothesized that amphipods will not significantly change the FR across MPP types. Assimilation efficiency (AE) as a biomarker describes that the test organisms function not only as an uptake agent but also as a resorption agent [24]. AE allows to assess how much material that is taken up is used for de-novo synthesis of consumer body tissue or energy storage. The biomarker wet weight change (WWch) measured after 28 days allows to assess whether MPP types have different effects on body mass gain or loss of the organisms. Previous work has shown that petroleum-based MPP lead to reduced growth in amphipods, potentially due to a longer residence time in the gut than natural food particles [25]. Amphipods produce digestive enzymes that allow for digestion of durable natural polymers such as lignocellulose [26,27]. We therefore hypothesized that the biodegradable MPP could be digested. Assimilation efficiency and WWch should then be less affected compared to exposure to the petroleum-based MPP. 

### 1.6. Natural Particles as a Realistic Double Control

In addition to testing and comparing the different MPP types against a control without any particles, we ran a second control using silica particles in standardized concentrations. Natural conditions in freshwaters are not particle-free. Freshwater shredders and filter-feeders such as amphipods and zooplankton should be well adapted to coping with the uptake of refractory materials [12]. Recent studies of MPP effects therefore included particles naturally occurring in the water column as a control (e.g., kaolin clay particles [12]). The silica particles used here are thought to represent naturally occurring particles for bottom-living freshwater organisms. Using this extra control with particles allowed us to indirectly detect whether MPP types differed in non-physical properties such as biochemical biodegradability. The silica control serves as a biologically inert benchmark. This benchmark allows for comparing the effects of mere particle-presence against physiological or toxic effects of MPP. 

## 2. Materials and Methods

### 2.1. Study Organisms

*G. fossarum* were collected in a brook (47°32′26″ N; 7°33′04″ E) near the city of Basel, Switzerland between April and June 2016. Only adult males passing a sieve with a mesh size of 3 mm, which corresponds to a cephalothorax length between 1.6 and 2 cm [28], were used. The amphipods were maintained in 10 L glass aquaria with a flow through of tap water (14 °C, 553 µS cm^−1^, oxygen saturation between 83% and 89%) and a light/dark cycle of 16 h/8 h. Amphipods were fed frozen chironomids ad libitum and pebbles were provided as shelter [24]. In total, 408 amphipods were used for the particle size ingestion- and excretion-experiment and 96 for the effect-experiment. 

### 2.2. Microplastic Particles Preparation

The pellets of both MPP types (Table 1) have a specific density higher than 1, allowing for the MPP to sink in the water column. Particles were powdered within a ball mill (MMR4000 Retsch GmbH, Haan, Germany) using liquid nitrogen to reduce the viscosity [29]. The powder passed through a sieve tower with 250 µm, 125 µm, 63 µm, 32 µm sieves to classify MPP in three different size ranges (32–63 µm, 63–125 µm, and 125–250 µm). For later identification of the MPP within the amphipod guts, Nile red stock solution β_Nile-red_ = 1 mg mL^−1^ was used for a 0.01% marking of the MPP, whereby for 100 mg MPP 10 µL Nile red stock solution was added to a test tube containing 5 mL of MilliQ water and after adding 50 µL of a SpanTween-surfactant (21% of Span20 and 79% of Tween80) the solution was cooked within a water bath for 60 min. After an ultrasonic bath for 5 min and exchange of fresh MilliQ water three times, the test tube was placed in the incubator for 24 h at 50 °C to dry the MPP. 

### 2.3. Food Preparation

As a standard substrate, we used decotabs which were specifically developed to assess the effects of plant, i.e., leaf litter quality on invertebrate consumption [30] and have been successfully applied in studying responses of the marine isopod *Idotea emarginata* to MPP [31]. They are liquefied in warm stages and solidified during cooling (solidification temperature approximately at 45 °C). For 1 L, 60 g cellulose powder (Sigma-Aldrich, St. Louis, MO, USA), 20 g agar (Sigma-Aldrich, St. Louis, MO, USA), and 60 µmol L-Ascorbic acid (105.6 mg, Sigma-Aldrich, St. Louis, MO, USA) were mixed and boiled. Standardized decotab discs were prepared for the effect experiment by pipetting 2.00–2.01 mg decotab material into aluminium trays using a balance (PI-214A, Denver Instruments, Bohemia, NY, USA).

### 2.4. Preparations Prior to Experiments

All amphipods were acclimatized at least 7 days prior to the experiments and individually starved for 48 h [32] prior to transfer into individual glass beakers (375 mL) containing 100 mL water, one decotab disc, and the specific MPP treatment (i.e., 10 to 100,000 particles individual^−1^). Particle numbers per individual were achieved by adding a certain volume of PHB/PMMA stock solution into the glass beaker using a pipette. The concentration of the stock solution as particles mL^−1^ had been previously determined (concentrations were 100,000 PHB particles per 13.2 mL volume of stock solution and 100,000 PMMA per 21.7 mL). When pipetting the solution into the beakers the volume was added such that MPP would sink equally distributed onto the bottom of the beaker. All treatment groups were run with 12 replicates: one individual amphipod per one of 12 glass beakers. The exposure time for each experiment was 24 h. After the experiment, living amphipods were euthanized by adding approx. 50 µL of clove oil. For the histological analysis the head still connected to the complete intestinal tract was separated from the pleon and parts of the pereon on a cover glass capped with several drops of PBS buffer (phosphate buffered saline, pH 7.4) and inspected under a fluorescent microscope (Nikon Eclipse E400, Nikon, Tokyo, Japan) to count the ingested MPP. 

### 2.5. Experimental Design

#### 2.5.1. Particle Size- and Concentration-Dependent Ingestion and Excretion Experiment

The particle size experiment tested the possible ingestion of different MPP size ranges (32–63 µm, 63–125 µm, and 125–250 µm). All treatment groups were run with 100,000 MPP individual^−1^ plus control without MPP. The particle concentration experiment was designed to determine the amount of ingested MPP in relation to increasing MPP concentrations (10, 100, 1000, 10,000, 100,000 MPP individual^−1^) and a control group without MPP [25,33]. The excretion experiment was designed to investigate the egestion capacities of the amphipods. The particle number (100,000 MPP individual^−1^) and the exposure time (24 h) was fixed and six different post exposure times for excretion were tested (1 h, 2 h, 4 h, 16 h, 32 h, and 64 h). The control in the excretion experiment consisted of individuals, which received particles in the same concentration but had no post exposure time to excrete the particles. After the exposure time, the amphipods were transferred to a new experimental beaker without MPP, which contained a new decotab disc for the specific post-exposure duration. 

#### 2.5.2. Effect Experiment

The effect experiment lasted for 28 days and contained two treatment groups (PHB and PMMA) and two control groups. One control group had no MPP and one had no MPP but silicon dioxide particles (Supelco Analytical, Munich, Germany; washed and calcined, puriss. p.a.). Silica particles were ground and sieved as the MPP to ensure the same size range (32–63 µm) which was identified as the most readily ingested particle size in the ingestion experiment (see Section 3). Silica was used to mimic naturally occurring mineral particles and thus provide a close approximation of natural MPP-free but not entirely particle-free conditions. The treatment groups were run with 24 replicates. The MPP concentration was fixed to 100,000 MPP individual^−1^. The amphipods were kept in a glass beaker, containing 300 mL of tap water, a standardized decotab disc (see food preparation) and the specific treatment. Each week the amphipods were transferred into new treatment or control beakers with identical conditions. Upon weekly transferal, the wet weight of the amphipods was measured by placing the amphipods with a stainless steel spoon on a blotting paper for 20 s to remove excess water and subsequently weighting them to the nearest 0.1 mg. The decotab of the previous week was placed on an aluminum tray, the egested feces pulled on a pre-dried and pre-weighted cellulose filter (Whatman, Maidstone, UK; qualitative filter papers, pore size 11 µm) using a vacuum pump, and both parts were dried in an incubator for 3 days at 50 °C prior to being weighted to the nearest 0.1 mg. Experiments were checked daily and molting or deceased animals were removed. 

**Figure 1:**
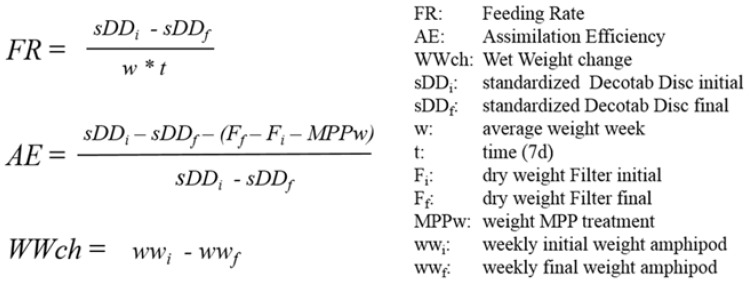
Equations of the studied biomarkers, feeding rate (FR), assimilation efficiency (AE) and wet weight change (WWch).

The three biomarkers (FR, AE, WWch) were measured according to standard protocols (Figure 1). Feeding rate was defined as daily ingested decotab material in relation to the wet weight of the amphipods multiplied with the feeding time, following previously published experimental work with natural substrates [24,28,34]. Assimilation efficiency was defined as the percentage ratio between assimilated and ingested decotab material, whereby the assimilated amount was calculated as the mass difference between the ingested decotab material and the egested feces in one week, subtracted by the weight of the MPP treatment, modified after published experimental work [24,28]. Wet weight change was defined as the weekly difference between the initial and final wet weight of the amphipods in one week, following previously published experimental work [22,35]. 

### 2.6. Statistical Analyses

For the ingestion and excretion experiment, the statistical analyses were performed with SPSS (Version 24 for Windows, SPSS Inc., Chicago, IL, USA). A Kruskal-Wallis test was used, due to a non-normal distribution of the data. Dunn-Bonferonni post-hoc tests were used for testing significance in differences between the treatment groups. For the effect-experiment, data was screened for normality (Shaprio-Wilk tests) and homogeneity of variance (Levene’s test, using the car package in R Studio). In the event that amphipods molted or died (only three instances), their individual data were excluded from the analyses for the respective incubation week. Across the experiments this led to the following number of excluded replicates: control (week one (w1) *n* = 6, w2 *n* = 4, w3 *n* = 3, w4 *n* = 3), silica (w1 *n* = 2, w2 *n* = 7, w3 *n* = 5, w4 *n* = 5), PHB (w1 *n* = 11, w2 *n* = 7, w3 *n* = 4, w4 *n* = 4), PMMA (w1 *n* = 1, w2 *n* = 12, w3 *n* = 11, w4 *n* = 4). If the ingested decotab disc weight was lower than 2.00 mg the replicate was also excluded from data analyses. This procedure followed previously published work which found that relative measurement error blurs the effects of extremely low ingestion and egestion masses [24]. To test for significant differences over the whole study duration, a two-way Repeated Measures Analyses of Variance (two-way RM ANOVA) with the main factors of treatment and time was used. The two-way RM ANOVA in SPSS gives information about whether there was a significant difference over time in the four experimental weeks (within-subject factor), as well as whether there was a significant difference between the four treatment groups (between-subject factor) summarized over all four weeks. However, this analysis cannot evaluate if there was a significant difference between one treatment group and another in a certain week. Therefore, independent pairwise t-tests with Benjamin and Hochberg correction for multiple tests were used. For the FR and WWch, Mauchly’s test for sphericity revealed a violation of assumptions, therefore Grenhouse-Geisser correction was applied prior to further analyses [36].

## 3. Results

### 3.1. Ingestion and Excretion Experiment

**Figure 2:**
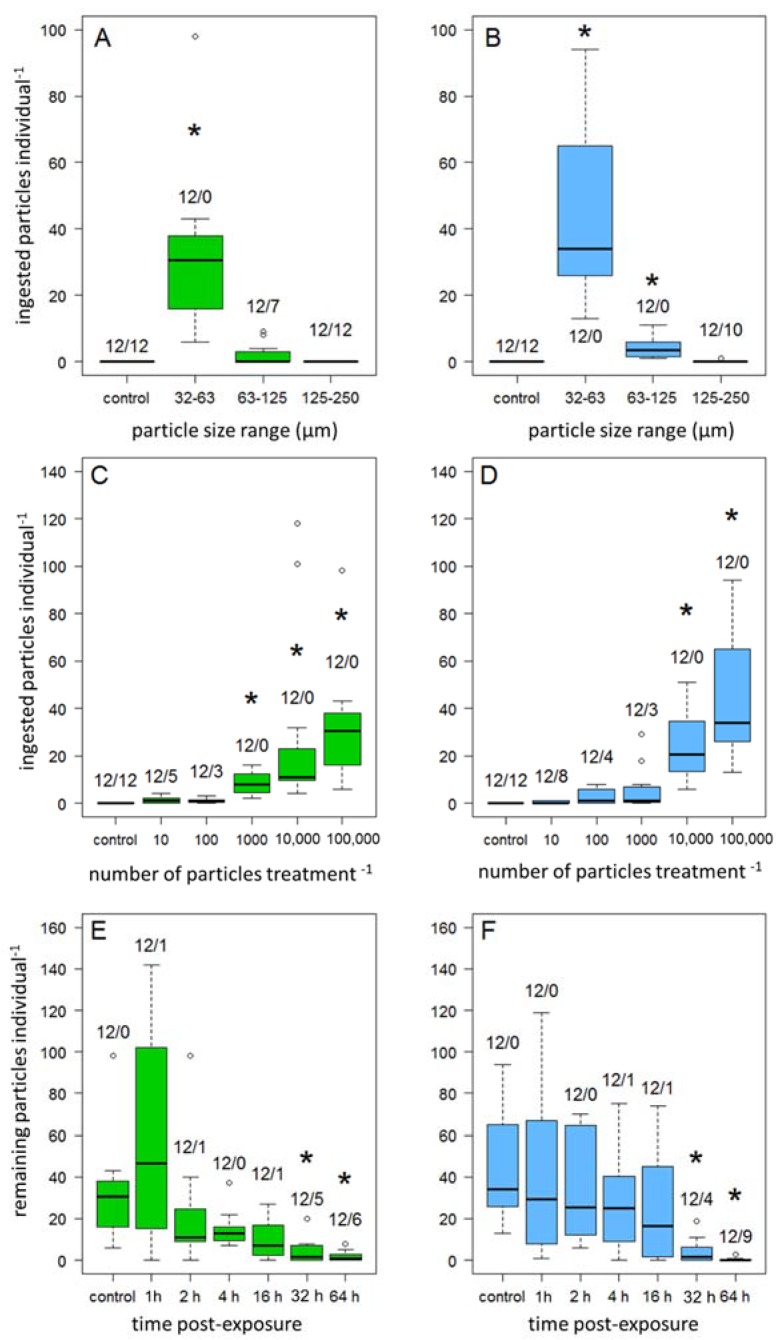
Amount of PHB (green) and PMMA (blue) particles detected in the digestive tract of amphipods. (**A**,**B**) Size experiment: the exposure concentration was fixed to 100,000 MPP individual^−1^ for 24 h for each size-range. (**C**,**D**) Concentration experiment: the exposure time was fixed to 24 h, the particle concentration depended on each concentration level (10–100,000 MPP individual^−1^). E/F Excretion experiment: after an exposure of 100,000 MPP individual^−1^ for 24 h, amphipods were placed in new beakers without MPP for the specific post exposure time (1 h, 2 h, 4 h, 16 h, 32 h, 64 h). XY/XY means: first number = replicates, second number = quantity of individuals with zero MPP in their guts, the control treatment had no post-exposure time. (**A**,**C**,**E**) PHB, (**B**,**D**,**F**) PMMA, Asterisk denotes significant differences at *p* < 0.05 compared with its control treatment.

Overall, there were no significant differences in ingestion and excretion between biodegradable and petroleum-based MPP. The size experiment showed that for both MPP types, the particles in size range 32–63 µm resulted in significantly higher ingestion (PHB/PMMA_32–63 µm_, *p* < 0.001; mean ingested MPP (±95% Confidence Interval, CI) (PHB_32–63 µm_ = 31.9 ± 15.2 *n* = 12/PMMA_32–63 µm_ = 45.6 ± 17.2 *n* = 12, Figure 2A,B). PMMA particles in the size range 63–125 µm also showed significantly higher ingestion than other particle size treatments (PMMA_63–125 µm_, *p* = 0.012, 4.1 ± 1.9, *n* = 12). Particles of size range 125–250 µm were not ingested, with the exception of two amphipods of the PMMA treatment. 

The concentration experiment showed a positive correlation (Spearman’s ρ = 0.89, *p* < 0.001, *n* = 72, Figure 2C,D) for both MPP treatments of the amount of ingested MPP with an increase of the provided MPP concentration. Significant differences between the concentrations and the control group were detectable in the PHB treatment at concentrations of PHB_1000_, *p* = 0.04, PHB_10,000_, *p* < 0.001, PHB_100,000_, *p* < 0.001, and in the PMMA treatment at concentrations of PMMA_10,000_, *p* < 0.001, PMMA_100,000_, *p* < 0.001. 

The excretion experiment showed significant decreases of ingested MPP after 32 h (PHB_32 h_, *p* = 0.018/PMMA_32 h_, *p* = 0.001) and 64 h (PHB_64 h_, *p* = 0.003/PMMA_64 h_, *p* < 0.001, see Figure 2E,F). The mean numbers of particles still remaining in the gut (±95% CI) within the post exposure time of 32 h were 4.0 ± 5.5 (*n* = 7) in the PHB and 4.1 ± 4.9 (*n* = 8) in the PMMA treatment. After 64 h post-exposure, 1.9 ± 2.7 (*n* = 6) of the PHB, and 0.4 ± 2.2 (*n* = 3) MPP in the PMMA treatment were found. 

### 3.2. Effect Experiment

#### 3.2.1. Feeding Rate

**Figure 3:**
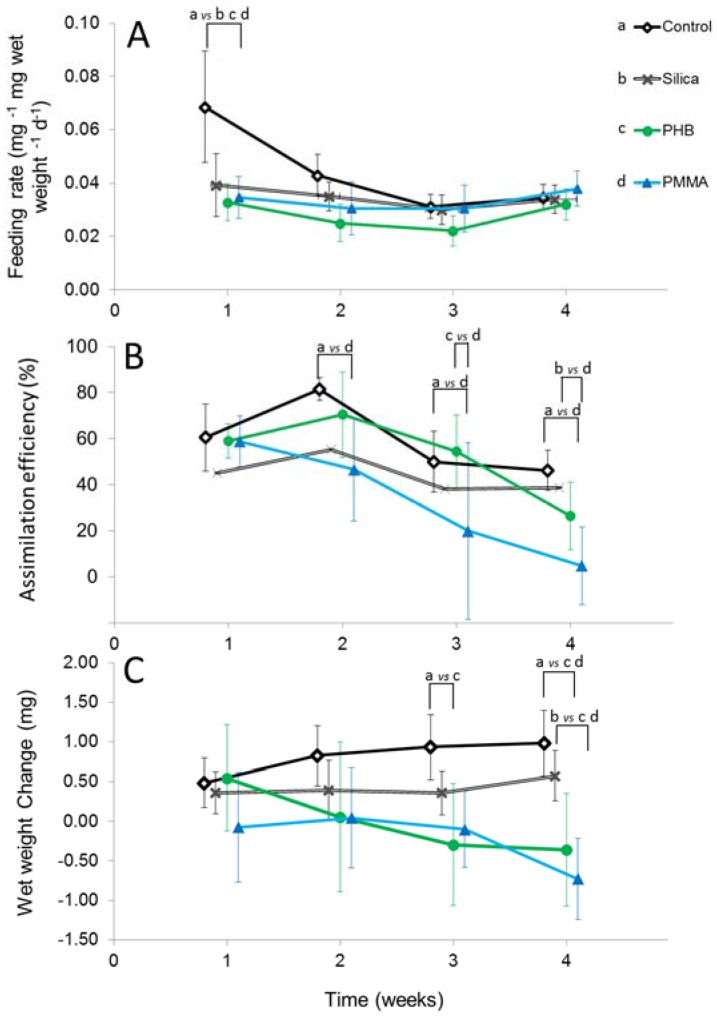
Weekly mean (±95% CI) of (**A**) Feeding rate; (**B**) Assimilation efficiency and (**C**) Wet weight change for control (w1 *n* = 18, w2 *n* = 20, w3 *n* = 21, w4 *n* = 21), silica (w1 *n* = 22, w2 *n* = 17, w3 *n* = 19, w4 *n* = 19), PHB (w1 *n* = 13, w2 *n* = 17, w3 *n* = 20, w4 *n* = 20), PMMA (w1 *n* = 23, w2 *n* = 12, w3 *n* = 13, w4 *n* = 20). Concentration fixed to 100,000 MPP individual^−1^. Feeding rate defined as the daily ingested decotab material mass in relation to the individual wet weight x days. Assimilation efficiency defined as percentage of the ratio between assimilated and ingested decotab material. Wet weight change defined as weight gain or loss after 7 days of exposure. For better visualization, the data points were staggered along the x-axis. Connected lines with letters denotes significant differences at *p* < 0.05 of pairwise tests, corrected for multiple comparisons.

The FR showed no significant differences over time of the four experimental weeks (RM ANOVA F_1.5,33_ = 2.6, *p* = 0.099), as well as no significant differences between the treatment groups (F_3,21_ = 1.2, *p* = 0.328). FR in the control treatment in the first week was significantly higher than in all other treatment groups, including the silica treatment (week 1 (w1) silica, PHB, PMMA vs. control, all *p* < 0.001) (Figure 3A). For the following three weeks, FR was similar for all treatments.

#### 3.2.2. Assimilation Efficiency

The AE showed a decrease across all treatment groups over the four experimental weeks (Figure 3B) RM ANOVA (F_3,63_ = 6.5, *p* = 0.001), and between the four treatment groups (F_3,21_ = 4.74, *p* = 0.011). The largest decrease over the duration of the experiment occurred within the PMMA group. Here, the mean (±95% CI) dropped from 58.8 ± 11.0% (*n* = 23) in week one to 4.8 ± 16.8% (*n* = 20) in week four (Figure 3B). Congruently, the AE in the PMMA treatment was different from the control treatment in weeks two (w2 PMMA vs. control, *p* = 0.024), three (w3 PMMA vs. control, *p* = 0.046), and four (w4 PMMA vs. control, *p* = 0.018). In week four the AE in the PMMA treatment was significantly lower also in comparison to the silica control (w4 PMMA vs. control, silica, *p* = 0.018, *p* = 0.013) (Figure 3B). The AE in the PHB treatment however, did not differ from any control. The only significant difference in the PHB treatments was found in comparison with the PMMA treatment: The AE in the PMMA treatment was significantly lower than that in the PHB treatment in week three (w3 PHB vs. PMMA, *p* = 0.023). 

#### 3.2.3. Wet Weight Change

The WWCh over time showed a marginal gain in weight in the control and silica treatments over the four weeks, as opposed to a weight loss in the PHB and PMMA treatment (Figure 3C). These trends over time are confirmed statistically (RM ANOVA F_1.5,31.4_ = 3.9, *p* = 0.042). Compared to the control, the WWch was signifcantly more negative in the PHB treatment in week three (w3 PHB vs. control, *p* < 0.001) and lower in both the PHB and the PMMA treatment in week four (w4 PHB, PMMA vs. control, all *p* < 0.001) (Figure 3C). In week four, WWch in the PHB and the PMMA treatment was significantly more negative compared to both the control and the natural silica particle control (w4 PHB, PMMA vs. silica, *p* = 0.036, *p* < 0.001) (Figure 3C). Amphipods in the PHB and PMMA treatment lost 0.36 mg (±0.52 95% CI) and 0.73 mg (±0.72) weight in week four, whereas amphipods from the control and silica treatment gained 0.98 (±0.41) and 0.57 mg (±0.32) in weight (Figure 3C). 

**Figure 4:**
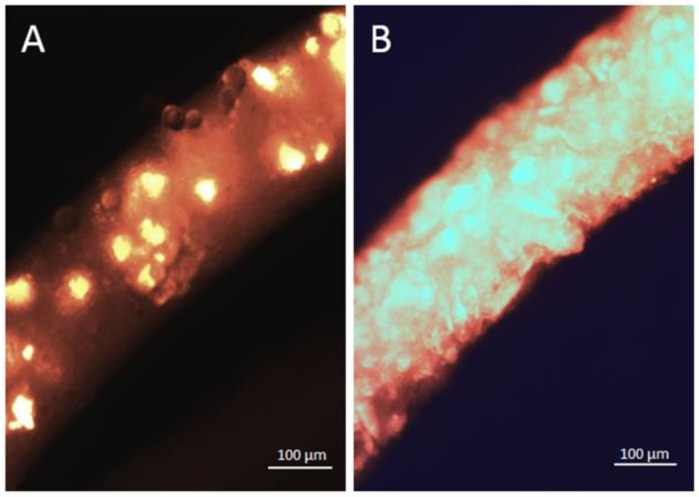

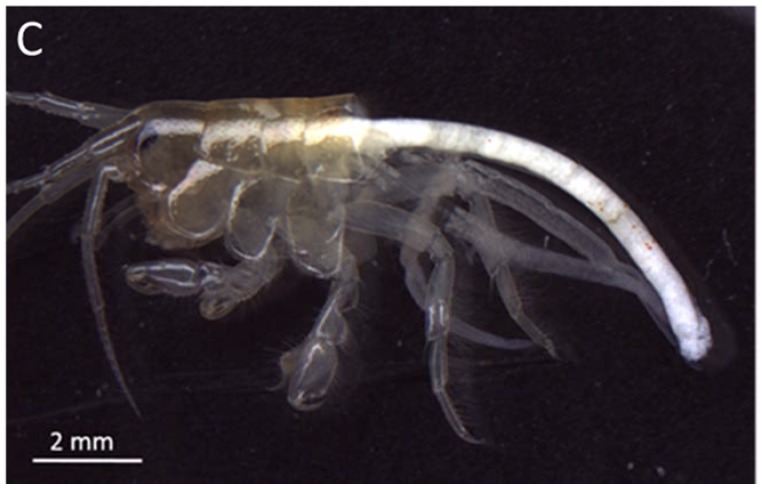
Dissected part of the gut of *Gammarus fossarum* containing ingested PHB particles. (**A**) Concentration experiment (100,000 MPP individual^−1^ 24 h^−1^ exposure time); (**B**) Effect experiment (100,000 MPP individual^−1^ 28 d^−1^ exposure time), bright spots/areas show fluorescent MPP; (**C**) A *Gammarus fossarum* specimen dissected for the analyses: the pleon and four segments of the pereon are removed such that mesenterium and caecum filled with opaque particles and decotab material are visible.

After the four experimental weeks, all amphipods of the four treatment groups were dissected according to the methods of the ingestion and excretion experiment (Figure 4). Within the MPP treatments, amphipods with a full gut (PHB *n* = 11, PMMA *n* = 10) showed such a high amount of MPP particles within their guts, that it could not be counted (Figure 4B). Mortality occurred twice in the PHB treatment (week one and four) and one amphipod in the silica control died in week four.

## 4. Discussion

Our findings suggest that biodegradable and petroleum-based MPP might both affect the ecologically relevant amphipods in freshwater ecosystems. The results of the ingestion and the excretion experiments and the patterns of the effect experiment indicate, that both biodegradable and petroleum-based MPP can be taken up by amphipods, which did not change their feeding rate in response to MPP presence. Biomarkers indicative of further challenges for the organisms showed significant effects of particles. There were overall few significant differences between the MPP types tested, but the petroleum-based MPP affected AE, whereas the biodegradable MPP did not. Wet weight change was negatively affected by the biodegradable MPP already after three weeks of exposure. After four weeks of exposure, the negative effects of both MPP types on WWch were detectable compared to the control, which contained natural silica particles. 

### 4.1. Ingestion

The highest average of ingested particles within the size range 32–63 µm of both MPP types matches previous work on MPP ingestion in amphipods. For instance, Au et al. [25] showed the ingestion of polyethylene beads (10–27 µm) and polypropylene fibers (20–75 µm length, diameter of 20 µm) in the freshwater amphipod *Hyalella azteca*. Overall, our study showed that the properties of both MPP types seem to be similar enough to result in the same size-selective ingestion. Most freshwater amphipods belong to the ecological feeding guild of shredders because they chew and grind larger food such as leafs into smaller particles. Shredding of larger MPP sizes into smaller ones, however, is unlikely given the hardness of the MPP types. The reason for the size-specific ingestion of particles might be related to the length and structure of the setae on the maxillulae capturing 32–63 µm large particles most efficiently. The size of the esophagus, which ultimately would limit ingestion, might further constitute a size selection factor [37].

### 4.2. Excretion

The excretion experiment showed that *G. fossarum* is able to excrete the MPP post exposure. After 32 h, significantly less MPP were found within the individual guts in contrast to the control group. The time for gut clearance of both MPP types appears rather long compared to previous studies. For example, natural leaf litter can be cleared from the gut by *Gammarus pseudolimnaeus* within 0.5 h to 2 h [38]. Experiments with PA fibres (20 × 500 µm) on natural food showed a nearly complete excretion after 4 h [22]. The longer duration until gut clearance in our study could be due to the use of decotab material. Decotabs have a generally longer gut-passage-time than natural leaf litter used in previous studies. The longer passage time might indicate that individuals had to cope longer with the particles than with natural food. Natural food would be faster to ingest and would have a faster gut passage than decotabs. Gut obstruction though accumulation of MPP has been put forward as one major effect of MPP on filter feeders [12]. In our study, the amphipods did not cease to feed when exposed to MPP (see below), which led to guts full of both decotab material and MPP. It is likely to assume this happening also in the field, where MPP become associated with natural food. It is plausible that gut retention time is a major pathway via which the different MPP affected amphipods in our study. 

### 4.3. Differences in Ingestion and Egestion between Microplastic Particles and Environmental Relevance of Concentrations

Overall, our results indicate that biodegradable MPP and petroleum-based MPP did not differ significantly in the way they are ingested or excreted. This suggests that organisms such as amphipods might not show different uptake patterns of both MPP types, as long as the physical properties of particles such as specific density are similar enough to allow for comparable encounter rates in the field. The high variance of the ingested MPP individual^−1^ might result from patchy distribution of MPP on the bottom of the experimental beaker, because we could not validate the exact distribution of particles on the bottom of the experimental beakers. Variation in individual feeding behaviour could also explain the discrepancy of the mean particle number of the 1 h post-exposure time and the control group within the excretion experiment. The lowest concentrations we offered are in a comparable range of what was reported in the environment, so our results are of high environmental relevance. The concentration of 10 and 100 MPP individual^−1^ with an experimental beaker bottom area of 20 cm^2^ result in a density of 5000 and 50,000 MPP m^−2^, which can well be found in aquatic environments. Although direct comparisons are difficult due to sampling and methodology differences, there are several studies, that make it reasonable to assume that we used environmentally relevant concentrations. For example, comparable concentrations have been recorded in the sediments of the Nakdong River Estuary in South Korea (up to 27,606 particles m^−2^) and in the sediments of the St. Lawrence River (maximum density of 140,000 microbeads >400 µm m^−2^) [39]. 

### 4.4. Feeding Rate

We hypothesized that amphipods would not significantly change the FR across MPP types. Our results support this hypothesis. Feeding rate was unaffected by any treatment in our study. FR in amphipods is a sensitive biomarker to detect effects of environmental pollution. Metal contaminations of *G. fossarum* significantly decreased the FR [40], as did the exposure of isopods to a neonicotinoid insecticide [23]. The lack of significant responses in FR in concert with the ingestion results suggest that none of the particles triggered an avoidance behavior. The fact that FR was unaffected by any particles indicates, that the feeding guild of shredders might readily take up particles of 32–63 µm size in freshwater systems. 

### 4.5. Assimilation Efficiency

We hypothesized that the biodegradable MPP should have less negative effects on AE because biodegradable material would be digested and thus be a source of energy. Our results only partly support this hypothesis. On average, the biodegradable MPP had no significant effect on AE, whereas the petroleum-based MPP negatively affected AE. Specifically, the petroleum-based MPP reduced the AE significantly compared to the control from the second week until the end of the experiment. Differences between MPP types were only significant in the third week. Previous studies comparing biodegradable and petroleum-based MPP did not find significant differences in how either MPP type affected feeding and filtration rates of marine organisms [13,14,41]. In our more controlled setting, we found that significant differences in AE were only detectable after three weeks and in comparison, with a particle-free control. In the fourth week however, the petroleum-based particle treatment had significantly lower AE than the silica particle treatment. This suggests that amphipod digestion remained unchallenged by the biodegradable MPP, albeit differences to the petroleum-based MPP are hard to detect within the chosen time frame. 

### 4.6. Wet Weight Change

More specific effects of MPP were visible only in the biomarker farthest ‘downstream’ of the uptake process, i.e., WWch. Individuals tended to lose weight in all treatments with MPP, whereas there was a tendency to gain weight in the control and in the silica treatment. The physical presence of the microplastic in the digestive tract generally leads to a reduced uptake of food, which in turn lowers energy intake with subsequent physiological effects [19]. The decotabs used here as a feeding baseline mostly consist of cellulose. Previous studies have found that not only energy content but also the presence of enzymes in the food influences AE in amphipods [42]. Ingestion and subsequent activity of digestive enzymes from fungi and bacteria growing as a biofilm on natural leaf litter the digestive capabilities in amphipods [26]. The absence of such enzymes or an insufficient biofilm formation in decotabs and silica particles might explain the rather low wet weight gain in the controls. A longer gut retention time, possibly leading to gut obstruction, might explain the negative effects on weight gain relative to the particle-free control. Such effects, if attributable to MPP, however, were significantly different from the silica control only after three weeks of exposure. This suggests that ingestion of non-palatable particles per se has no negative effect on amphipods. The presence of MPP in the gut, however, had more effects on WWch than the presence of natural silica particles, if we assume the latter were ingested. These results indicate effects of MPP which go beyond the mere physical presence. Our findings thus provide more mechanistic understanding in support of previous studies, which found both biodegradable and petroleum-based MPP to affect marine organisms [13,14,41]. In summary however, the assumption that biodegradable MPP will have less biological effects must be rejected based on the grounds of insignificant differences in WWch between MPP types. The explanation for this could be that gut obstruction is a negative effect of both biodegradable and petroleum-based MPP, though not of natural particles.

### 4.7. Effects of Biobased and Biodegradable Plastics on Organisms

Amongst the most important distinction between the MPP types tested here, is the rate and ability of biodegradation of bioplastics versus a high durability of petroleum-based plastics in the environment. The potential biological effects of biodegradable plastics on organisms are relatively poorly studied [13]. We know even less about whether the effects of particles are in any way contingent on the biodegradability. The particles of two MPP types were comparable in their physical properties such as mass density, sphericity, overall shape, and size. Any differences in effects, or the lack thereof, are thus plausible to result from factors other than physical properties. The rate of biodegration is the most plausible explanation for expecting differences between the MPP types. Yet, our results suggest that effects on WWch did not signficiantly differ among MPP types at the concentrations and timescales we applied. Recent research applied long-term experiments of 8 month duration to detect strong negative effects of chronic MPP exposure on FR, body mass, and nutritional status in the langoustine *Nephrops norvegicus* [43]. Biodegradation of biodegradable MPP bags in the gastrointestinal fluid of sea turtles, Green turtle (*Chelonia mydas*) and a Loggerhead turtle (*Caretta caretta*), resulted in a total mass loss of ingested bags up to 9% within 49 days [44]. These studies suggest that long term experiments are needed to detect effects of MPP and that biodegradable plastic could indeed be digested by aquatic consumers. Biodegradation is tested under standardized industrial biodegradation conditions, with a defined temperature which is typically higher than ambient temperatures in nature. Still, the few existing studies suggest that biodegradation might also occurr under natural environmental conditions. To what extent the conditions in the digestive tract of aquatic organisms lead to appreciably faster degradation rates requires further study.

### 4.8. Challenges to the Digestive System of Benthic Consumers—The Importance of Microbiota

The digestive tract in *Gammarus* spp. has been studied from a morphological and enzymatic perspective and a complex interplay between ingested and endogenously produced enzymes has been described [26,27,42]. Digestive enzymes and the presence or absence of microbiota and biofilms both in the gut and the environment can greatly affect an individual’s ability to digest both biodegradable and petroleum-based MPP. For example, it has recently been demonstrated in polystyrene-digesting mealworms (*Tenebrio molitor* larvae), that microbiota in the gut allow for efficient assimilation of polystyrene [45]. The question whether biodegradable plastic might indeed be less inert than petroleum-based MPP in a consumer’s gut is a promising avenue for further research. An important benchmark against which to test such effects are controls featuring natural particles. Nature is not particle free and freshwater organisms are exposed to a variety of particles depending on e.g., the catchment’s sediment load, so any realistic test of artificial particles should appreciate the ubiquitous presence of natural particles. The epipsammic, i.e., growing on sand, biofilms are important for the trophic ecology of bottom-living freshwater organisms [46]. All particles, including the silica particles, in the experiment were in water at ambient temperature for several hours prior to consumption. This time might have well allowed for differential biofilm formation across different particle types [47]. This in turn, might have affected to what extend the ingested particles constitute a challenge to the digestive system. Despite the similar shape of particles, there might be differences between biodegradable and petroleum-based particles in the way the surface is colonized by microbiota. This would have ramifications for AE and WWch because of the important role that biofilms play for digestion in amphipods. The importance of biofilms for physiological effects of MPP should be appreciated in future experimental studies on MPP effects on invertebrate consumers. Including natural particles into such experiments will also shed light on when and how particle consumption in nature actually poses a physiological challenge.

## 5. Conclusions

In conclusion, our hypotheses that biodegradable MPP would have appreciably less physiological effects on freshwater amphipods were only partly supported, whereas our hypotheses that both petroleum-based and biodegradable MPP would be readily ingested in certain sizes was supported. 

To expand on our results, we need more field monitoring on the relative occurrence of both biodegradable and petroleum-based particles in nature. Whether and how the actual property of biodegradability, which did not seem to affect the uptake, at all mediates the negative effects of MPP remains an important question for future research. Standards for biodegradation are developed for industrial composting or soil conditions. Against this background it is clear that certification of biodegradable plastic is not informative of actual behavior under natural freshwater conditions. Biodegradable plastics entering the aquatic environment would not nearly encounter the conditions of standardized biodegradation assays. To advance the development of environmentally friendly alternatives to petroleum-based plastics, we need a better understanding of the biological fate of biodegradable MPP in the field. 

## Figures and Tables

**Table 1:** Overview of polyhydroxybutyrate (PHB) and polymethylmethacrylate (PMMA) microplastic particle (MPP) properties and visual comparison of particles under the microscope.

MPP	PHB	PMMA
Density	1.24 g cm^−3^	1.19 g cm^−3^
Color	white, opaque	transparent
Form before grinding	pellets (3 mm)	pellets (3 mm)
MPP sizes after grinding	32–64 µm	32–64 µm
	64–125 µm	64–125 µm
	125–250 µm	125–250 µm